# Heat stress directly impairs gut integrity and recruits distinct immune cell populations into the bovine intestine

**DOI:** 10.1073/pnas.1820130116

**Published:** 2019-05-07

**Authors:** Franziska Koch, Ulrike Thom, Elke Albrecht, Rosemarie Weikard, Wietje Nolte, Björn Kuhla, Christa Kuehn

**Affiliations:** ^a^Institute of Nutritional Physiology “Oskar Kellner,” Leibniz Institute for Farm Animal Biology (FBN), 18196 Dummerstorf, Germany;; ^b^Institute of Genome Biology, FBN, 18196 Dummerstorf, Germany;; ^c^Institute of Muscle Biology and Growth, FBN, 18196 Dummerstorf, Germany;; ^d^Faculty of Agricultural and Environmental Sciences, University Rostock, 18059 Rostock, Germany

**Keywords:** heat stress, infiltrating immune cells, jejunal mucosa, laser capture microdissection, RNAseq

## Abstract

High ambient temperature has multiple potential effects on the organism such as hyperthermia, endotoxemia, and/or systemic inflammation. However, it is often difficult to discriminate between cause and consequence of phenotypic effects, such as the indirect influence of heat stress via reduced food intake. Lactating dairy cows are a particularly sensitive model to examine the effects of heat stress due to their intensive metabolic heat production and small surface:volume ratio. Results from this model show heat stress directly induced a so-far unknown infiltration of yet uncategorized cells into the mucosa and submucosa of the jejunum. Due to a pair-feeding design, we can exclude this effect being a consequence of the concurrent heat-induced reduction in feed intake. Isolation and characterization of the infiltrating cells using laser capture microdissection and RNA sequencing indicated a myeloic origin and macrophage-like phenotype. Furthermore, targeted transcriptome analyses provided evidence of activated immune- and phagocytosis-related pathways with LPS and cytokines as upstream regulators directly associated with heat stress. Finally, we obtained indication that heat stress may directly alter jejunal tight junction proteins suggesting an impaired intestinal barrier. The penetration of toxic and bacterial compounds during heat stress may have triggered a modulated immune repertoire and induced an antioxidative defense mechanism to maintain homeostasis between commensal bacteria and the jejunal immune system. Our bovine model indicates direct effects of heat stress on the jejunum of mammals already at moderately elevated ambient temperature. These results need to be considered when developing concepts to combat the negative consequences of heat stress.

## Significance

Consequences of heat stress, particularly for the immune system and the intestinal health of mammals, are a topic of increasing global relevance due to rising temperatures and potential health impairments. Specific climate effects, however, are often difficult to discriminate from indirect consequences, e.g. reduced feed intake. Our study in dairy cattle, which are particularly sensitive to heat, identifies the infiltration of the small intestinal epithelium by a previously unobserved distinct cell population with macrophage-like phenotype in response to moderate heat stress. By using a pair-feeding design, we attributed these effects as direct consequences of heat stress via impaired intestinal barrier function. Therefore, an appropriate gut function is an important component in combating the negative consequences of heat stress.

Global warming is associated with an increased risk of extreme heat events as characterized by temperatures exceeding the long-term averages of magnitude, frequency, and duration (1). Mammals with intensive metabolic heat production and a relatively small surface:volume ratio (Bergmann’s rule) are particularly prone to heat stress due to limited capability for heat dissipation. To accommodate radiant heat dissipation through the body surface, the blood flow increases toward the periphery while it is reduced in the splanchnic area (2, 3). The resulting hypoxia diminishes ATP, induces reactive nitrogen species production, and modulates gut integrity (4).

A tight intestinal barrier, as formed by the interaction of tight junction proteins (TJPs) of adjacent enterocytes, protects the host against paracellular bacterial infiltration and penetration of toxic substrates such as endotoxins, digestive enzymes, and degraded food products (5, 6). The loss of intestinal barrier integrity allows for the paracellular transport of endotoxins [e.g., lipopolysaccharide (LPS)] into the blood stream leading to the activation of the innate immune system and systemic inflammation (4). A typical intestinal inflammatory response after LPS entering the submucosa involves various immune cells located in the lamina propria or Peyer’s patches (7, 8). While dendritic cells recognize pathogen components via their pattern recognition receptors and trigger differentiation of T and natural killer cells, monocytes, Kupffer cells, and macrophages may secrete proinflammatory cytokines (9).

In patients with inflammatory bowel disease (IBD) developing a “leaky gut,” macrophages massively infiltrate the intestinal mucosa in response to the invasion of bacteria and endotoxins (10, 11), whereas in patients with Crohn’s disease, macrophages enter into the muscular layer of the gut (12) and mesenteric fat (13). However, which immune cells participate in the immune response of the intestine during heat exposure has not been investigated.

Heat-stressed humans and rats have elevated plasma concentrations of proinflammatory cytokines (14, 15). Increased gut IL-1β, IL-6, and TNFα concentrations enhance intestinal cell permeability by increasing claudin and reducing occludin and zonula occludens 1 (ZO-1) protein expression (16), but whether the local cytokine abundance changes with heat stress is not known. Claudins, occludin, and junctional adhesion molecules are the major transmembrane proteins forming a selective paracellular barrier, whereas ZO-1–3 are main cytoplasmic proteins located at the inner cellular membrane (17). Heat stress but not pair feeding at thermoneutrality has been shown to increase *occludin*, *ZO1*, and *claudin 3* mRNA abundance in the intestine of growing pigs (18), but no protein data were presented. Furthermore, it is not known whether the immune response reported after heat challenge is due to the ambient heat itself or due to the associated decline in feed intake. Therefore, the objective of this study was to elucidate a potential immune cell response specifically to long-term heat stress (>24 h) and to characterize the intestinal cytokine profile, oxidative stress response, and gut barrier integrity in the jejunum in comparison with pair feeding at thermoneutrality.

## Results

### Intestinal Morphology and Immune Cells.

**Fig. 1.**
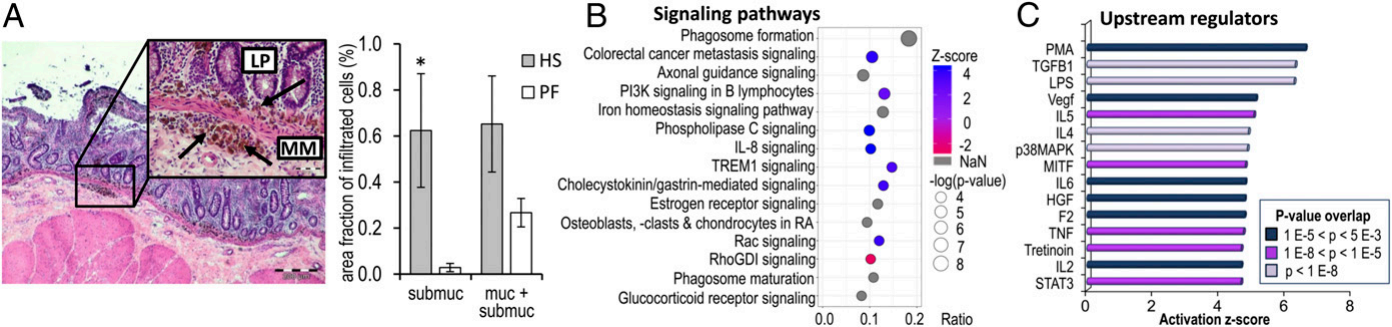
Heat stress induces intestinal inflammation. (*A*) Representative image of infiltrating cells in the lamina propria (LP) and underneath the muscularis mucosae (MM) after 4 d of heat stress (H/E staining). (Scale bar, 200 μm.) (*Inset*) High magnification. (Scale bar, 50 μm.) Area percentage of infiltrating cells in the submucosa and mucosa + submucosa of HS and PF cows. *P < 0.05; *n* = 5 (means ± SEM). (*B*) Top 15 canonical pathways and (*C*) upstream regulators of genes significantly more highly expressed in infiltrating cells in the jejunum of HS cows compared with whole jejunum mucosa of age-matched control cows.

Jejunum morphology as examined by villus height and crypt depth was not significantly different between heat-stressed (HS) and pair-fed (PF) cows (*SI Appendix*, Fig. S1). However, HS cows showed a distinct clustering and an increased number of infiltrating cells of unknown lineage in the submucosa striking by higher optical density (Fig. 1*A* and *SI Appendix*, Fig. S2*A*). The infiltrating cells covered a significantly larger fraction of jejunal submucosa sections in HS compared with PF cows (*P* = 0.042; Fig. 1*A*). While these cell populations were also observed in the jejunal mucosa (*SI Appendix*, Fig. S2*B*), no difference in their distribution was observed between HS and PF cows.

### RNA Sequencing-Based Holistic Expression Profile of Infiltrating Cells.

To identify lineage and characteristics of the cell population which infiltrated into the submucosa of HS cows, tissue areas containing those cells were excised by laser capture microdissection (LCM) (*SI Appendix*, Fig. S2*C*) and were subjected to whole ribo-depleted RNA sequencing (RNAseq). The global transcriptome profiles from four samples of the excised cells were determined and their enriched biological pathways were compared with controls from whole jejunal mucosa of age-matched cows kept at thermoneutrality.

The expression patterns of all four infiltrating cell samples displayed a high conformity with very similar fragments per kilobase million (FPKM) values across samples (Datasets S1 and S2 genes with >5 FPKM and 10-fold expression). Comparing the global transcriptome of the LCM samples and samples from whole jejunum mucosa of age-matched cows revealed 3,076 significantly differentially expressed genes (q < 0.05), of which 1,052 genes were more highly expressed in infiltrating cells and 2,024 genes were more highly expressed in the jejunal mucosa (Dataset S3). These differential expression data are in very good agreement with an initial comparison between the infiltrating cells and a whole jejunum mucosa sample, which was processed using the same ultra-low input library preparation method as with the LCM samples (*SI Appendix*). The list of the 2,024 genes with higher expression in the jejunal mucosa of control cows compared with HS cows comprised a large number of genes known to be specific to or very highly expressed in intestinal epithelial cells (e.g., *MGAM*, *ENPEP*, *SI*, *KRT8*, *KRT19*, *CLDN7*, *CLDN15*, and *SLC27A4*). Ingenuity pathway analysis (IPA) of those 2,024 genes showed an enrichment of genes involved in oxidative phosphorylation, mitochondrial dysfunction, sirtuin signaling pathways, cholesterol biosynthesis, and EIF2 signaling. In contrast, IPA of those 1,052 genes significantly more highly expressed in the infiltrating cells of HS cows indicated a significant enrichment of genes involved in phagosome formation, colorectal cancer metastasis signaling, axonal guidance signaling, PI3K signaling, and iron homeostasis pathway (Fig. 1*B* and Dataset S4). It is noticeable that the list of these genes comprise several highly and differently expressed immune defense-associated genes, i.e., *SLC11A1* encoding the NRAMP1 protein and *SLC40A1* (involved in iron metabolism and host resistance to certain pathogens), *CD68* (member of the scavenger receptor family clearing cellular debris, promoting phagocytosis and mediating the recruitment and activation of macrophages), *ABCA9* (induced during monocyte differentiation into macrophages), *NOD1* (pattern-recognition receptor), and *CPM* (associated with monocyte-to-macrophage differentiation). This list of genes more highly expressed in infiltrating cells also includes *LYZ*, known to have activity against numerous bacterial species in tissues and body fluids associated with the monocyte/macrophage system. Furthermore, genes encoding lysosomal proteinases, such as cathepsins (e.g., *CTSK*, *CTSB*, *CTSD*, and *CTSZ*) are enriched in the infiltrating cells. Cathepsins are known to be involved in digestive processes, indicating enhanced proteolytic activity of phagocytosis, endocytosis, and tissue remodeling (19).

Within the top 15 activating upstream regulators predicted by IPA, we found TGFB1 and MITF, IL6, IL4, TNF, and LPS, all supporting the immunological characteristics of infiltrating cells as obtained from the canonical pathways enrichment analysis (Fig. 1*C* and Dataset S5). For TGFB1 and MITF, our RNAseq expression data directly confirmed a significantly higher transcript expression in infiltrating cells compared with whole jejunum mucosa as predicted from IPA.

**Fig. 2.**
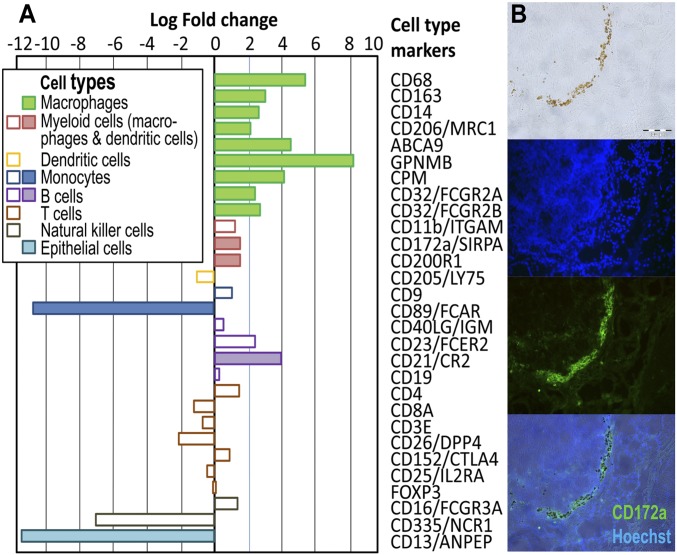
Identification of infiltrating cells in the jejunum of HS cows. (*A*) Differential gene expression levels of marker genes specific for particular cell types or immune functions. Color-filled bars: significant fold changes, q < 0.05; open bars: nonsignificant fold changes. Negative/positive value indicates lower/higher expression in infiltrating cells from HS cows compared with whole jejunal mucosa of age-matched cows. (*B*) Fluorescent image of infiltrating cells detected with an antibody against CD172a, marker for dendritic cells, and macrophages [brightfield, Hoechst 33258 (blue), CD172a positive cells (green), merged image]. (Scale bar, 100 µm.)

Focusing on marker genes known to be associated with specific immune cell types or immune functions in our dataset, we identified 14 of 29 selected genes with differential expression levels in infiltrating cells of HS cows compared with jejunum mucosa. Among them, 12 genes were significantly more highly expressed and 2 were lower-expressed genes (Fig. 2*A* and *SI Appendix*, Table S1). The higher expressed genes include macrophage/phagocyte/myeloid cell-associated genes like *CD68*, *CD163*, *CD14*, *MCR1* (*CD206*), and *FCGR2B* (*CD32*), indicating that infiltrating cells are of macrophage-like cell type. In contrast, genes characteristic for B cells or T cells (e.g., *CD4*, *CD8A*, *CD3E*, and *CD40LG*), natural killer cells or dendritic cells [*FCGR3A* (*CD16*), and *LY75* (*CD205*)] did not show differential expression levels between infiltrating cells of HS cows and control mucosa.

### Immunohistochemical Confirmation of Macrophage-Like Cell Identity.

To confirm the identity of the infiltrating immune cells, parallel sections of the tissue used for LCM were subjected to immunohistochemistry. Single CD3^+^ and CD21^+^ cells were found in villi (*SI Appendix*, Fig. S3 *A* and *B*), not within the cluster of infiltrating cells. Instead, infiltrating cells were immune positive for CD172a and CD163 (Fig. 2 and *SI Appendix*, Fig. S4), which corresponds to the higher respective gene expression levels in these cells compared with whole jejunal mucosa. The antibody against CD172a stained exclusively the infiltrating cells (Fig. 2 *C* and *D*), whereas CD163 was also apparent in other cells (*SI Appendix*, Fig. S4 *C* and *D*). CD172a encoded by *SIRPA* is reported to be particularly abundant in cells from the myeloid lineage, such as macrophages and dendritic cells (20), whereas CD163 is exclusively expressed in monocytes and macrophages (21, 22). Thus, we conclude that infiltrating cells are primarily a subtype of the macrophage phenotype.

### Tight Junction Proteins.

**Fig. 3.**
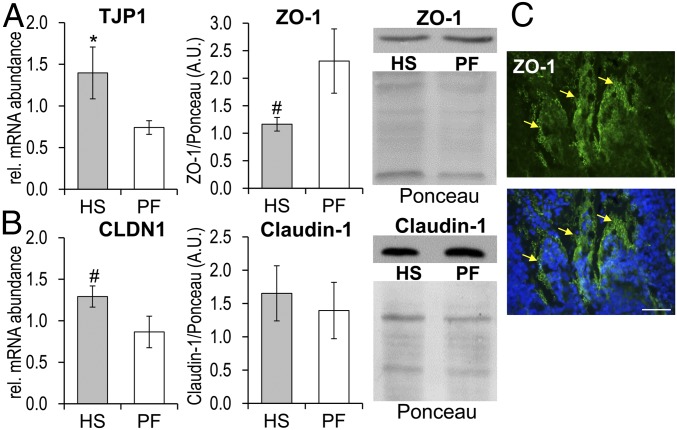
Heat stress induces alterations of TJPs abundance in jejunal mucosa. (*A*) *TJP1* mRNA and protein abundance (ZO-1) with one representative Western blot for HS and PF cows. (*B*) *CLDN1* mRNA and protein abundance with one representative Western blot for HS and PF cows. Data were normalized to total protein after Ponceau staining. (*C*) Immunohistochemical staining of the jejunum using an antibody specific for ZO-1. (Scale bar, 50 µm.) *n* = 5 (means ± SEM). *P < 0.05 and ^#^0.05 < *P* < 0.1.

To investigate whether the jejunal immune response is associated with impaired gut barrier function during thermal stress, targeted expression analysis of selected genes encoding tight junction proteins was performed in the jejunal mucosa of HS and PF cows. Analysis of RT-qPCR data revealed 2-fold higher *zonular occludens 1* (*TJP1* encoding ZO-1) mRNA abundance in HS compared with PF animals (*P* = 0.05; Fig. 3*A*). *Claudin 1* (*CLDN1*) mRNA abundance showed a trend with 1.5-fold higher expression in HS cows (*P* = 0.096; Fig. 3*B*), while the other tight junction proteins investigated were similar in both cow groups (*SI Appendix*, Fig. S5). Western blot analysis of corresponding proteins showed that ZO-1 protein abundance tended to be lower in HS cows (1.5-fold, *P* = 0.096; Fig. 3*A*) and was predominantly localized in the membranes of epithelial cells (Fig. 3*C*), whereas claudin-1 abundance was similar to that in PF cows (Fig. 3*B*).

### Immune and Acute Phase Response.

**Fig. 4.**
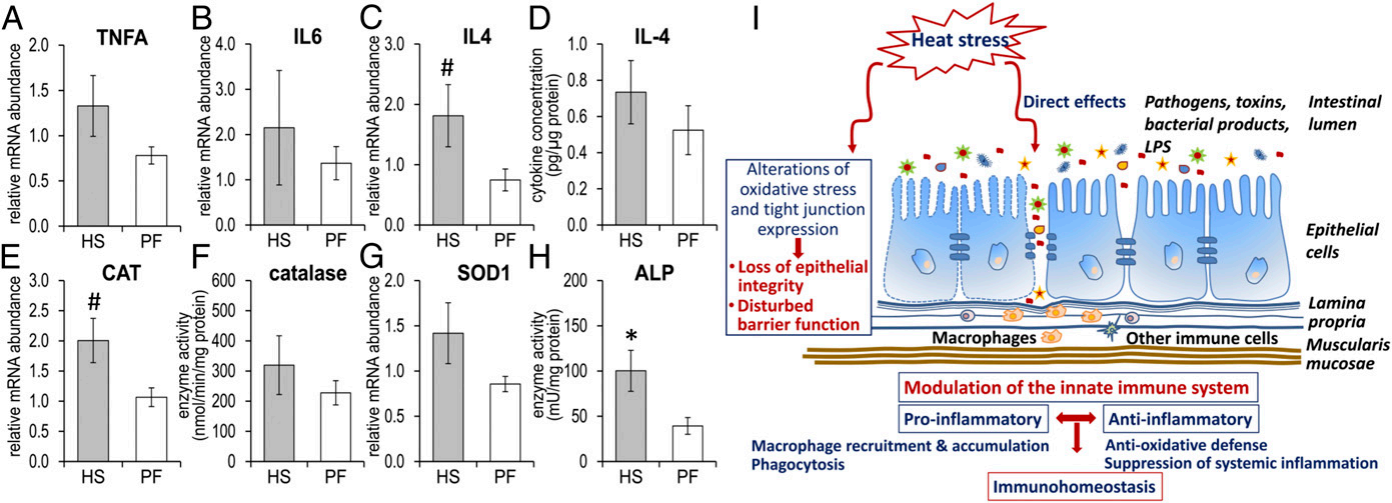
Higher *IL4* and *CAT* gene abundance and ALP activitiy in mucosa after long-term heat stress. RT-qPCR analysis of the mRNA expression of (*A*) *TNFA*, (*B*) *IL6*, (*C*) *IL4*, and (*D*) IL4 protein after 4 d of heat stress or pair feeding. (*E*) mRNA analysis of CAT and (*F*) catalase enzyme activity, (*G*) mRNA expression of *SOD1*, and (*H*) ALP activity in jejunal mucosa of HS and PF cows. *n* = 5 (means ± SEM). *P < 0.05 and ^#^0.05 < *P* < 0.1. (*I*) Model of direct long-term heat-stress effects on the jejunal mucosa in lactating dairy cows.

We next examined gene expression of selected specific markers for intestinal stress, inflammation, and immune defense in the whole jejunal mucosa of HS and PF cows. The results showed that *TNFA*, *IL6*, *IL10*, *CXCL5*, and *haptoglobin* (*HP*) mRNA expression was not significantly different between HS and PF cows (Fig. 4 *A* and *B* and *SI Appendix*, Fig. S6 *B*–*D*). The *IL4* mRNA expression representing an antiinflammatory response tended to be higher in HS compared with PF animals (2.4-fold, *P* = 0.096; Fig. 4*C*), whereas IL-1β and IL-4 protein levels showed no difference between both groups (Fig. 4*D* and *SI Appendix*, Fig. S6*A*).

### Oxidative Stress Defense.

To evaluate potential damage on cell membranes caused by thermal stress, marker genes/proteins associated with oxidative stress were examined in the jejunum mucosa of HS and PF cows. Catalase (*CAT*) mRNA abundance tended to be 2.2-fold higher in HS compared with PF cows (*P* = 0.095; Fig. 4*E*). However, the mRNA abundances of genes encoding other antioxidative enzymes, such as *superoxide dismutase 1* (*SOD1*) and *glutathione peroxide 1* (*GPX1*), were similar between HS and PF cows (Fig. 4*G* and *SI Appendix*, Fig. S6*E*). Catalase and lysozyme activities were not different between groups (Fig. 4*F* and *SI Appendix*, Fig. S6*F*), but the activity of alkaline phosphatase (ALP) was significantly higher in the mucosa of HS than PF cows (*P* = 0.05; Fig. 4*H*).

## Discussion

Heat stress has been shown to disrupt tight junctions in the intestine thereby increasing intestinal permeability in rodents, pigs, and humans (4, 18, 23). The resulting increased penetration of bacteria, small particles, and toxic compounds elicit an inflammatory response in the gut (5, 6, 24). However, the (immune) cells participating in the adaptation of the intestine to environmental heat and whether these effects are directly caused by the heat stress or by the associated reduction in feed intake are unknown (25). We compared pair-fed dairy cows kept at thermal neutral conditions with heat-stressed animals constantly kept at 28 °C for 4 d and investigated the morphological and functional responses in the jejunum epithelium.

### Heat Stress Triggers the Migration of Infiltrating Cells into the Mucosa and Submucosa While Jejunal Morphology Remains Unaltered.

While we did not find differences in villi or crypt morphology in response to heat stress, histological analysis revealed clusters of infiltrating cells of an unknown cell type both in the mucosa and submucosa, primarily in HS animals. Thus, heat stress triggers the migration of infiltrating cells into the mucosa and submucosa while the jejunal morphology remains unaltered compared with PF cows. Pigs kept constantly at 35 °C for 7 d had decreased villi heights and crypt depths relative to thermal neutrally kept (nonpair-fed) counterparts, but had comparable villi heights to PF pigs (18). These and our results indicate that villi height is not specifically controlled by heat stress, but rather affected by reduced energy and nutrient intake.

### Functional and Immunohistochemical Portrait of Infiltrating Cells Indicates Macrophage-Like Phenotype.

The most important signaling pathway overrepresented in the transcriptome of infiltrating cells was phagosome formation in conjunction with phagosome maturation. Results of the IPA analysis suggest an activated phagocytosis, which is an essential mechanism of the innate immune response to maintain tissue homeostasis (13). Professional phagocytes, such as macrophages, neutrophils, monocytes, and dendritic cells, are known to be particularly engaged in phagocytosis. Further enriched pathways in heat stress-induced infiltrating cells were linked with phagocytosis and macrophage activation/activity: the Rac signaling pathway, the immune-related pathways known to trigger the activation and survival of myeloid cells, the phospholipase C signaling, and the maintenance of iron homeostasis. It has been reported that the Rac signaling pathway is involved in phagocytosis and the elimination of invading pathogens and/or toxic compounds (26). Activation of immune-related pathways, such as IL-8, TREM1, and glucocorticoid receptor signaling, are known to trigger the activation and survival of myeloid lineage (27, 28), strongly indicating that macrophage-like cells dominate the cluster of infiltrating cells in the submucosa of HS cows. Phospholipase C signaling is involved in phagocytosis, represents a signal transduction pathway in macrophage polarization (29), and is important in macrophage-mediated inflammatory response (30). The maintenance of iron homeostasis in infiltrating cells seems to play a pivotal role in the response to heat stress. This is supported by the substantially enriched expression of genes associated with iron homeostasis, such as *SLC40A1* (*ferroportin*) and *SLC11A1* (*NPAMP1*), which are involved in ionic iron transport and metabolism and host resistance to certain pathogens, as well as *FTH1* and *FTL* encoding subunits of ferritin, the major intracellular iron storage protein. Free heme detoxification, provision of iron for de novo hemoglobin synthesis, and establishing the balance in heme iron metabolism are some of the most important metabolic tasks of macrophages (31). In humans, inflammatory disorders of the gastrointestinal tract such as IBD are characterized by disturbances in iron homeostasis, including abnormal intracellular sequestration of iron in monocytes and a decrease in circulatory iron concentrations (32). In our study, a decrease in hematocrit was found in HS cows (33). However, whether heat stress leads to hypoferremia is not known.

Positive myeloid marker CD172a staining and a corresponding significantly higher expression of the *SIRPA* gene encoding CD172a in the infiltrating cells compared with the control mucosa confirmed the classification of the infiltrating cells as macrophage cell type. Macrophages comprise a heterogeneous population. Tissue-resident macrophages in the intestine account for about one-fifth of all leukocytes (34, 35) and can be distinguished by their localization: between macrophages located directly underneath the epithelium and those residing centrally in the lamina propia, above the muscularis mucosae (10). Our study reports infiltrating immune cells in the submucosa after heat stress. The expression of *CD14* in infiltrating cells supports the view that some of these cells originate from blood recruited monocytes and terminally differentiate into macrophage-like cell subtypes. In contrast, resident macrophages have no CD14^+^ phenotype in normal and healthy mucous membranes (36).

When intestinal epithelial integrity is impaired, e.g., during infection or inflammatory challenge, blood monocytes are recruited as macrophage precursors to actively track invading microorganisms, release inflammatory mediators, and scavenge apoptotic cells and foreign molecules (36). Macrophage infiltrations have also been detected in human intestine in necrotizing enterocolitis (NEC) (37), in the intestinal mucosa in IBD and throughout the thickened mucosa and submucosa in patients with Crohn’s disease (10). However, the unique phenotype and functional profile of bovine intestinal macrophages are not yet known. Even the precise phenotypic and functional patterns of human and murine intestinal macrophage subpopulations are still under debate (38). Therefore, in this study a specific focus was made on the expression profiles of marker genes encoding products belonging to the cluster of differentiation (CD). This targeted analysis highlighted 14 immunospecific genes in infiltrating cells of HS cows with a different expression compared with control mucosa. The majority of the higher expressed genes, including *CD14*, *CD68*, *CD163*, *MCR1* (CD206), *FCGR2B* (CD32), *CPM*, *GPNMB*, and *ABCA9*, are known to be predominantly related to the CD and transcriptional signature of macrophages. From the RNA expression analysis, the presence of regulatory T and natural killer cells in the infiltrating cell clusters was excluded as *FOXP3* and *NCR1* (CD335) expression was not detected. However, it cannot be excluded that in addition to macrophages, further immune cell populations have infiltrated the mucosa and submucosa, since mRNA expression was observed for marker genes specifically expressed by B cells (*CD21*, *CD23*), and monocytes (*CD9*). However, expression of *FCAR* (CD89), also a monocyte indicator, was significantly higher in the control whole jejunum mucosa, advocating against monocyte enrichment in the infiltrating cells. While the expression data may suggest marginal contribution of B cells, lack of CD3 and CD21 immunohistochemical signals in the infiltrating cell population argues against the presence of B and T cells. However, CD3^+^ and CD21^+^ cell types were observed in the villi with similar numbers in HS and PF cows.

Among upstream regulators responsible for the activation of signaling pathways in infiltrating cells, distinct cytokines (IL5, IL4, IL6, IL2), TGFB1, and LPS were identified. It can be hypothesized that LPS, a constituent of bacterial cell walls, enters the mucosa more distinctively during heat stress and activates the immune response via toll-like receptors (TLRs). This is supported by a thermotolerance study in bovine blood, demonstrating that TLR 2/4 and IL 2/6 play an important role in adapting to the effects of short- and long-term heat exposure (39).

### Indication of Impaired Intestinal Barrier Due to Heat Stress as Background for Immune Cell Infiltration in the Jejunal Mucosa.

Heat stress was found to affect TJPs and intestinal integrity in rats and Caco-2 cells (2). The TJPs are gate guards and border protectors, formed by ZOs, claudins, occludins, and junctional adhesion molecules (16), which allow the passage of ions through paracellular pores (40). Our results indicated higher *CLDN1* and *TJP1* mRNA abundance in HS cows in line with findings in pigs, in which *TJP1* but also *CLDN3* mRNA abundance increased after 7 d of heat exposure (18). These alterations of TJPs were related to linear transepithelial electrical resistance reduction, increased LPS permeability, and increased endotoxin, indicating gut leakiness after 7 d of heat stress in pigs (18). The latter study also showed that the *TJP* mRNA abundance was not affected when the exposure to heat lasted for only 3 d (18). Thus, the similar duration of heat exposure applied in our study may explain why no significant differences in *MLCK*, *TJP2*, and *OCLN* mRNA abundances were found.

Our data indicates that stress-induced activation of the mucosal defense system elicited by ALP takes place in HS cows. The latter plays a key role in intestinal detoxification of LPS, CpG DNA, and flagellin by prevention of bacterial translocation to the intestine and suppression of the inflammatory response to proinflammatory factors (41, 42). ALP expression and activity is influenced by numerous stressors and dietary components, leading to changes in detoxification mechanisms and suppression of systemic inflammation in the intestine (43). This study reports up-regulation of ALP activity in response to heat stress in the intestine of HS cows. In humans, decreased ALP levels were observed in some pathological conditions in the intestine due to impaired inhibition of inflammation, but the available data do not support a direct link of ALP to intestinal barrier function (44). Nevertheless, ALP has potential in the prevention and treatment of the consequences resulting from a disturbed intestinal barrier function, as observed in metabolic syndrome and gut-derived systemic inflammation (45).

In Fig. 4*I*, we propose a summarizing model of direct long-term heat-stress effects on the jejunal mucosa, particularly the innate immune system, based on the data obtained in our experiment. Due to the pair-feeding regime of our animal model, our data indicate that heat stress may directly lead to an increased infiltration of cells from the adaptive immune system into the lamina propria and underneath the muscularis mucosae of the jejunum. This effect is heat-stress specific and independent of the associated reduction in feed intake. Isolation and characterization of these infiltrating cells by LCM and RNAseq showed a specific gene expression pattern characterizing the activation of phagocytosis and immune-related pathways elicited by LPS and cytokines. Furthermore, distinct CD expression signatures pointed toward a predominant accumulation of macrophage-like cells. The infiltration of immune cells may occur due to altered junction connections and impaired intestine barrier function at high ambient temperatures. Pathogens, bacterial compounds, and small particles, e.g., LPS, can enter the epithelial border and trigger an inflammatory response via TLRs, leading to an activation of the antiinflammatory cytokine IL-4, an induction of the antioxidative defense machinery (e.g., CAT) as well as protective suppression of systemic inflammation by ALP. Thus, it can be concluded that long-term heat stress may directly trigger balancing mechanisms in the jejunum to maintain homeostasis between commensal bacteria and the immune system.

## Materials and Methods

*SI Appendix*, *Supplementary Material and Methods* provides additional detail.

### Animals and Experimental Design.

Ten German Holstein cows in second lactation (HS: week 28 ± 8; PF: week 39 ± 16; *P* > 0.3) were adapted to the climate chamber at thermoneutral conditions [15 °C, 63% relative humidity (RH), temperature humidity index (THI) 60] for 6 d. Five animals were allocated to HS at 28 °C (52% RH, THI 76, ad libitum feeding) or PF at 15 °C (63% RH, THI 60, restrictive feeding according to feed intake of HS cows), respectively. After 4 d of HS or PF, jejunum samples and jejunum mucosa scrapings were taken and stored at −80 °C for analysis. For whole transcriptome expression analyses, we included a jejunum whole mucosa sample from a 80-d-old calf essentially as described in ref. 46 as well as jejunum samples from four age-matched lactating control cows in their second lactation, fed ad libitum a total mixed ration and kept at thermoneutrality (details in ref. 47). All procedures were approved by the ethics committee of the State Government in Mecklenburg-West Pomerania, Germany (LALLF M-V/TSD/7221.3–1.1–074/12, LALLF M-V/TSD/7221.3–2.1–010/03) and the local department for animal welfare affairs (Landesuntersuchungsamt, Koblenz, Germany, 23 177–07/G 13–20-069).

### Intestinal Histology and Image Analysis.

Tissue sections were cut with a cryostat microtome (Leica CM3050S), stained with hematoxylin and eosin, and analyzed with Cell^D image analysis software (OSIS) as detailed in *SI Appendix*.

### LCM.

Infiltrating cells in the jejunum of HS cows were collected with LCM for subsequent identification. Cryosections, 12 µm thick, were cut (CM 3050S, Leica), transferred to PALM membrane slides (PALM), and dehydrated. Cells were cut and collected in adhesive caps (PALM) with a PALM MicroBeam LCM device.

### RNA Isolation.

Total RNA was isolated from the whole jejunal mucosa scrapings essentially as described (46) and for the infiltrating cells captured by LCM, the RNeasy Micro Kit (Qiagen) or the Nucleo Spin RNA XS Kit (Macherey-Nagel) was used according to the manufacturer’s specifications.

### Library Preparation for LCM-Based and for Global Jejunal Mucosa RNAseq.

Four global stranded RNAseq libraries from the infiltrating cells captured by LCM and one from whole jejunal calf mucosa total RNA (positive control for low RNA input) were prepared using NuGen’s Ovation SoLo RNA-Seq Kit (NuGen Technologies) according to manufacturer recommendations. Stranded libraries from jejunum mucosal total RNA were prepared from four age-matched control cows (kept at thermoneutrality) using the TruSeq Stranded Total RNA Ribo-Zero H/M/R Gold Kit (Illumina). All RNAseq libraries were sequenced in a multiplex design on the Illumina HiSeq2500 platform with 2 × 100 bp paired-end sequencing cycles. Additional details are provided in *SI Appendix*.

### RNAseq Bioinformatic Analyses.

The processed reads from the infiltrating cells and the jejunum whole mucosa libraries were mapped to the NCBI bovine reference genome assembly UMD3.1 using HISAT2 (v2.0.3, ref. 48) with the Ensembl *Bos taurus* genome annotation v87. Details of data processing and bioinformatic analysis are provided in *SI Appendix*. Gene expression was quantified by the featureCounts algorithm from the Subread package (v1.5.2, ref. 49). The global transcriptome profile of infiltrating cells of HS cows sampled by LCM was compared with data from jejunal whole mucosa of age-matched control cows, for differential expression analysis (50). Lists of genes significantly more highly expressed in either the infiltrating cell samples or in the control cow whole jejunum mucosa were separately submitted to enrichment analyses via IPA (Qiagen Inc., https://www.qiagenbioinformatics.com/products/ingenuity-pathway-analysis) (51) to identify overrepresented pathways in both counterparts.

### Immunohistochemistry.

Markers for specific immune cell populations were selected for immunohistochemical analysis, such as CD3, CD21, CD163, and CD172a. Tissue sections were cut 10 µm thick with a cryostat microtome, fixed with 4% paraformaldehyde in PBS, and incubated with respective antibodies as detailed in *SI Appendix*. Nuclei were counterstained with Hoechst 33258 (Sigma-Aldrich). TJPs were detected using an antibody against ZO-1 (GTX108592 Genetex). Fluorescence was visualized with a Nikon Microphot SA fluorescence microscope (Nikon) and an image analysis system equipped with CELL^F software and a CC-12 high-resolution color camera (OSIS).

### RT-qPCR.

RNA of jejunum mucosa samples was extracted by RNeasy Mini Kit (Qiagen). First strand cDNA synthesis was performed using random primers, dNTPs, RNase inhibitor, reverse transcriptase, and 5× Buffer Revert Aid (Thermo Fisher Scientific). Transcriptional expression was quantified by RT-qPCR on LightCycler 96 (Roche) with primers (*SI Appendix*, Table S2) and SensiFAST SYBR No-ROX mix (Bioline). Data analysis was performed with LinRegPCR software (v2014.4) and qbase software (Biogazelle).

### Western Blot.

Western blot analysis of mucosal jejunum samples was performed by SDS/PAGE and transferred to membrane. For the detection, primary antibodies against zonula occludens 1 (ZO-1, H-300 Sc-10804, Santa Cruz Biotechnology) and claudin-1 (sc-166338, Santa Cruz Biotechnology) were utilized and the corresponding secondary antibodies (goat anti-rabbit IgG HRP, sc-2004; Santa Cruz Biotechnology or rabbit anti-mouse IgG HRP, AS09 627, Agrisera AB). Chemiluminescent reagents (Pierce ECL Western Blotting Substrate, Thermo Fisher Scientific) were applied, and blots were exposed to hyperfilms (GE Healthcare). Hyperfilms and Ponceau S-stained membranes were scanned and individual bands or lanes, respectively, were quantified using ImageJ (v1.49).

### Jejunal Inflammation and Stress Markers.

Mucosa samples were analyzed for lysozyme activity (EnzChek Lysozyme Assay Kit; Thermo Fisher Scientific), alkaline phosphatase activity (QuantiChrom Alkaline Phosphatase, BioAssay Systems), catalase activity (Cayman Chemicals), and interleukin-1β and interleukin-4 protein, utilizing specific ELISA (bovine anti–IL-1β, ESS0027, bovine anti–IL-4, ESS0031; Thermo Fisher Scientific).

### Statistics.

Group effects were analyzed using the nonparametric tests and parametric, Mann–Whitney *U* test or Student’s *t* test, including the UNIVARIATE procedure of SAS (v9.4; SAS Institute Inc.). Data are given as mean ± SE (SEM). Results were considered as statistically significant at *P* < 0.05 and trends between 0.05 < *P* < 0.1.

## Supplementary Material

Supplementary File

Supplementary File

Supplementary File

Supplementary File

Supplementary File

Supplementary File
