# Supplementary material for: Heat stress directly impairs gut integrity and recruits distinct immune cell populations into the bovine intestine
Source: Proc Natl Acad Sci U S A. 2019 May 7;116(21):10333–8. doi: 10.1073/pnas.1820130116 (PMC6535017; doi:10.1073/pnas.1820130116)
Supplement: Supplementary File [file pnas.1820130116.sapp.pdf]

PNAS
www.pnas.org

Supplementary Information for

Heat stress impacts on bovine intestinal immune system: identification of infiltrating immune cells following impaired gut integrity

Franziska Koch, Ulrike Thom, Elke Albrecht, Rosemarie Weikard, Wietje Nolte, Björn Kuhla, Christa Kühn

Elke Albrecht
Email: elke.albrecht@fbn-dummerstorf.de

This PDF file includes:

Supplementary Material and Methods
Figures S1 to S6
Tables S1 to S2
Captions for databases S1 to S5
References for SI reference citations

Other supplementary materials for this manuscript include the following:

Datasets S1 to S5

www.pnas.org/cgi/doi/10.1073/pnas.1820130116

Supplementary Material and Methods

Animals and experimental design

Ten German Holstein cows were genotyped for HSP70.1 5′UTR 895 (reference sequence M98823.1; (1)). Three different genotypes were identified and equally assigned to heat-stressed (HS, n = 5) or pair-feeding (PF, n = 5) group. Pair feeding served as a control to consider the main effect to heat stress, because HS cows showed reduced feeding behavior (2). All cows were in established 2nd lactation (HS: week 28 ± 8; PF: week 39 ± 16; P > 0.3). Animals were adapted to the climate chamber at thermoneutral conditions (15°C) for six days and received a total mixed ration twice daily at 0700 h and 1500 h (3). As described recently (4), five HS cows were subsequently exposed for four days to 28 °C with 52 ± 2 % relative humidity (RH) resulting in a temperature-humidity index (THI) of 76 with *ad libitum* feeding. Cows had free access to water, feed and water tempered to 28 °C. The reduction of daily *ad libitum* intake of HS cows was calculated as percentage of the mean daily feed intake to provide the same amount of feed energy to PF cows under thermoneutral conditions. The five PF cows were exposed for four days to 15 °C with 63 ± 1 % RH and THI of 60. After four days, rectal temperature of HS or PF amounted to 40.2 and 38.4 °C, respectively. Animals were sacrificed in the institutional slaughterhouse, and jejunum samples and jejunum mucosa scrapings were taken, frozen in liquid nitrogen and stored at -80 °C until later analysis. For histological analyses, tissue samples were punched out from the jejunum wall, embedded in Tissue-Tek (Sakura Finetek, Zoeterwoude, Netherlands) and stored at -80 °C. For whole transcriptome expression analyses, we included a jejunum whole mucosa sample from a 80 days old calf essentially as described in Hammon et al. (5), as well as jejunum samples from four age-matched lactating control cows in their second lactation, fed *ad libitum* a total mixed ration and kept at thermoneutrality (details in Weikard et al. (6)). All procedures were approved by the ethics committee of the State Government in Mecklenburg-West Pomerania, Germany (LALLF M-V/TSD/7221.3-1.1-074/12, LALLF M-V/TSD/7221.3-2.1-010/03) and the local department for animal welfare affairs (Landesuntersuchungsamt, Koblenz, Germany (23 177-07/G 13-20-069).

Intestinal histology and image analysis

Embedded frozen tissue was cut (4 µm thick) on a cryostat microtome (Leica CM3050S, Bensheim, Germany). Three sections from each animal were stained with hematoxylin and eosin (H/E; Hematoxylin, Dako, Germany; Eosin, Chroma Gesellschaft, Münster, Germany) according to standard procedures and visualized by using an Olympus BX43 microscope (Olympus, Hamburg, Germany) equipped with a UC30 color camera and Cell^D imaging software (OSIS, Münster, Germany). Ten villi heights and ten crypt depths per sample section (magnification 10x) were measured with the interactive measurement module of Cell^D software comparable to the procedures described by Pearce et al. (7). The average of 30 villi heights and crypt depths were calculated and reported as one number per cow. The closed polygon function within the interactive measurement module of Cell^D software was used to measure the area of submucosa and infiltrating cells in all available H/E stained sections. The area fraction of infiltrating cells was calculated. The measurement of infiltrating cells in mucosa, *Muscularis mucosa* and submucosa was performed in unstained sections. A macro program was developed to

obtain repeatable results. After loading the image, the green channel was extracted and a region of interest (ROI) was defined, comprising the submucosa, *Muscularis mucosa*, and mucosa within the image. Then, a threshold was set to detect infiltrating cells. False detected particles could be deleted interactively. The area of the ROI and all detected cells were measured in ten images per sample. The area fraction was calculated from the total area of ROIs measured and the area occupied by infiltrating cells.

Laser capture microdissection (LCM)

Infiltrating cells in the jejunum of HS cows were collected with LCM for subsequent identification according to the procedure basically as described by Albrecht et al. (8). Cryo-sections, 12 µm thick, were cut (CM 3050S, Leica), transferred to PALM membrane slides (PALM, Bernried, Germany) and air-dried for 10 s. Sections were dehydrated by placing the slides in different concentrations of ethanol (1 x 70 %, 1 x 96 %, 2 x 100 %) for 30 s each and in xylene for 5 min. Finally, the sections were air-dried under a fume hood for 5 min. Cells were cut and collected in adhesive caps (PALM) with a PALM MicroBeam laser capture microdissection device. The total cut areas were 145.6 µm², 128.0 µm², 23.1 µm², and 96.2 µm² for samples LCM1, LCM2, LCM6, and LCM7 (Datasets S1-S3), respectively. Assuming an average cell size of 150 µm² (measured in immunofluorescence images), the estimated cell number in each sample corresponded to 970, 850, 150, and 640 cells, respectively. The actual number was approximately 10 % lower because of loss of particles during catapulting into the adhesive caps.

RNA isolation

RNA isolation from the whole jejunal mucosa scrapings was performed essentially as described by Hammon et al. (5). RNA concentration was determined via Qubit analysis (Thermo Fisher Scientific, Berlin, Germany). RNA integrity was checked via Bioanalyzer 2100 (Agilent Technologies, Waldbronn, Germany) and potential DNA contamination according to Weikard and colleagues (9). Total RNA of the infiltrating cells isolated by LCM was prepared and purified using either the RNeasy Micro Kit (Qiagen, Hilden, Germany) or the Nucleo Spin RNA XS Kit (Macherey-Nagel, Düren, Germany) according to the manufacturer's specifications and stored at -80 °C until further processing.

Library preparation for LCM-based RNAseq and for global jejunal mucosa RNAseq

Four RNAseq libraries were prepared from the infiltrating cells isolated by LCM using NuGen's Ovation SoLo RNA-Seq Kit (NuGen Technologies, San Carlos, CA, USA). According to manufacturers' recommendations, a qPCR step was performed on the LightCycler 96 (Roche Diagnostics, Mannheim, Germany): prior to the final library amplification, the number of PCR cycles optimal for amplification was determined. Finally, all libraries were amplified using 19 cycles before being sequenced. In addition to the four LCM-based RNAseq libraries of infiltrating cells, a RNAseq library from 100 pg calf whole jejunal mucosa total RNA was prepared with the same protocol used for the dissected infiltrating cells to serve as positive control for low RNA input and for subsequent comparison of gene expression between dissected infiltrating cells and jejunal

mucosa. The final five RNAseq libraries were analyzed for correct size distribution with an Agilent Bioanalyzer 2100 (Agilent Technologies, Waldbronn, Germany), quantified using a Qubit Fluorometer (Invitrogen, Carlsbad, CA, USA) and sequenced in a multiplex design on the Illumina HiSeq2500 platform using 2x100 bp paired-end sequencing cycles and standard Illumina primers.

Comparing the LMC samples and the whole jejunum calf mucosa sample processed with the same library preparation method, 215 genes showed an expression above 5 FPKM in all LCM samples and an average expression across all LCM samples exceeding 10-fold the expression level in the control mucosa (Dataset S2). Among the 215 genes identified in the initial numerical analysis, 155 were also contained within the 3,076 significantly ($q < 0.05$) differentially expressed genes identified when comparing the LCM samples and the whole jejunum mucosa samples of the age-matched cows demonstrating a very good agreement between analyses.

Analogous to the RNAseq libraries generated for the infiltrating cells (NuGen's Ovation SoLo RNAseq), stranded libraries from the entire jejunum mucosal RNA were prepared from four control cows age-matched to the HS cows and kept at thermal neutrality to establish a reference profile of regular tissue from unchallenged animals. Libraries were prepared using the TruSeq Stranded Total RNA Ribo-Zero H/M/R Gold Kit (Illumina, San Diego, USA). This protocol also included a ribo-depletion step, but did not select for polyA-tailed molecules. Quality control and sequencing of the resulting libraries was performed as described above for libraries generated using NuGen's Ovation SoLo protocol.

RNAseq bioinformatic analyses

The resulting raw reads from NuGen's Ovation SoLo RNAseq and whole jejunum mucosa Illumina Ribo-Zero RNAseq were demultiplexed, converted into FASTQ file format with Illumina's bcl2fastq2 conversion software (v2.17.1.14) and checked for quality using the FastQC tool (v0.11.5; (10)). Prior to the alignment, we removed adapter sequences and for the NuGen's Ovation SoLo generated reads also the first 5 bp of the forward reads as recommended in NuGen's manual using cutadapt (v1.13; (11)). In addition, subsequent quality trimming applying qualtrim removed low quality reads (https://bitbucket.org/arobinson/qualitytrim). Finally, the processed reads from the infiltrating cells as well as for the jejunum whole mucosa were mapped to the NCBI bovine reference genome assembly UMD3.1 using HISAT2 (v2.0.3; (12)) in the downstream transcript assembly mode (dta) with the Ensembl *Bos taurus* genome annotation v87 together with the strand-specific option and visualized using the Integrative Genomics Viewer (v2.4.7; (13, 14)). Gene expression was quantified from the generated bam files by the featureCounts routine from the Subread package (v1.5.2; (15)) applying the Ensembl *Bos taurus* genome annotation v87 and by subsequent in-house R scripts.

As a first step comparing gene expression between samples processed with the same NuGen's Ovation SoLo protocol, genes were filtered for FPKM values of at least 5 and a 10-fold higher average expression in all infiltrating cell samples compared to the low input control mucosa. In addition to the initial tentative expression analysis between infiltrating cell samples and a jejunal whole mucosa low input control sample, all processed with the identical library preparation method, a second control data set was

included for final differential expression analysis. This second control data set comprised the global transcriptomic ribo-depleted non-polyA selected reads from jejunal whole mucosa of four cows age-matched with the HS cows from which the infiltrating cells had been sampled by LCM. We used raw read counts as determined by featureCounts with the edgeR package (16) to determine potential differences in gene expression between infiltrating jejunal cells of HS cows and jejunum whole mucosa of age-matched control cows. The Biomart platform (https://www.ensembl.org/info/data/biomart/index.html) transferred Ensembl identifiers to gene names. In addition, Ensembl identifiers without gene name assignment in Biomart were manually assigned gene names according to the NCBI *Bos taurus* UMD3.1.1 annotation (accession 2018-04-28). Lists of genes significantly higher expressed in the infiltrating cell samples and genes significantly higher expressed in the cow jejunum mucosa were separately submitted to enrichment analyses via Ingenuity Pathway Analysis (IPA, Qiagen Inc., https://www.qiagenbio-informatics.com/products/ingenuity-pathway-analysis, (17)) to obtain information about overrepresented pathways. High purity of the collected cells was confirmed by RNAseq data demonstrating that markers of epithelial cells in the small intestine e.g. CD13/ANPEP (18) or genes known to be highly and specifically expressed in small intestinal mucosa (*FABP6*, https://gtexportal.org/home/gene/FABP6, (19)) displayed no expression in isolated cells (Dataset S1). However the respective genes were substantially transcribed in whole mucosa tissue (Dataset S3).

Immunohistochemistry

For immunohistochemical analysis, CD3, CD21, CD163 and CD172a were selected as markers for specific immune cell populations. Antibodies directed against these markers had been demonstrated to recognize the proteins of bovine origin (20-23). Tissue sections were cut 10 µm thick, fixed for 15 min with 4 % paraformaldehyde in PBS and washed. Sections were incubated with either Alexa Fluor 488 labeled rat anti-human CD3 (1:50, AbD Serotec) or FITC labeled mouse anti-human CD21 antibody (1:10, AbD Serotec) at RT for 1 h in a humidity chamber. Slides were washed 3 x 10 min in PBS and nuclei were counterstained with Hoechst 33258 (1:10,000 in PBS; Sigma-Aldrich, St. Louis, MO, USA). After washing, slides were covered using ProLong Diamond Antifade Mountant (Thermo Scientific) and appropriate cover-slips. Fluorescence was visualized with a Nikon Microphot SA fluorescence microscope (Nikon, Tokio, Japan) and an image analysis system equipped with CELL^F software and a CC-12 high resolution color camera (OSIS). The number of $CD3^+$ and $CD21^+$ cells was counted in ten randomly selected villi and the respective villus areas were measured. The number of positive cells divided by the area of the villus (cells/mm^2) was determined for each animal. Additionally, CD163 and CD172a antibodies were utilized to characterize infiltrating cells. Tissue sections were cut 10 µm thick with a CM3050S cryostat microtome (Leica), air dried for 15 min, fixed with 4 % paraformaldehyde in PBS, and washed 3 x 5 min in PBS. Unspecific binding of the secondary antibody was blocked using 10 % goat serum in PBS for 15 min. Slides were incubated for 1 h at room temperature (RT) with either mouse anti-human CD163 (1:50, AbD Serotec, Puchheim, Germany) or CD 172a (1:10; (21)), and subsequently washed 3 x 10 min with PBS. Specific binding of the primary antibodies was detected with the respective Alexa Fluor 488 goat anti-mouse IgG (Thermo Fisher Scientific, Waltham, MS, USA) or MFP 488 goat anti-mouse IgG

(Mobitec, Göttingen, Germany) secondary antibody (1:500, 45 min at RT in the dark). Slides were washed 3 x 10 min in PBS and nuclei were counterstained with Hoechst 33258 (1:10,000 in PBS; Sigma-Aldrich). After washing 2 x 5 min with PBS and 1 x 5 min with distilled water, slides were covered using ProLong Diamond Antifade Mountant (Thermo Scientific) and appropriate cover-slips.
Tight junction proteins were detected using antibodies against ZO-1 (1:50, GTX108592 Genetex) and ZO-2 (1:50, sc-515115 SantaCruz) following the protocol described before, except that both antibodies were incubated at 4°C overnight. Specific binding of the primary antibodies was detected with an Alexa Fluor 488 goat anti-rabbit IgG (Thermo Fisher Scientific) and a MFP 488 goat anti-mouse IgG (Mobitec) secondary antibody (1:500, 45 min at RT in the dark), respectively.

Reverse-transcriptase quantitative PCR (RT-qPCR)
Jejunum mucosa samples were mortared under liquid nitrogen and total RNA was extracted from 100 mg tissue powder with TriFast Reagent (Peqlab, Erlangen, Germany) and cleaned with RNeasy Mini Kit (Qiagen). The RNA quality was determined with an Agilent 2100 Bioanalyzer (Agilent Technologies). The RIN factors for mucosa were between 5.9 and 7.9 (mean: 7.25). First strand cDNA synthesis (375 ng RNA) was completed using 250 pmol random primers (Metabion International, Planegg/Steinkirchen, Germany), 10 mM dNTPs each, 20 U RNAse Inhibitor, 200 U Revert Aid, and 5x Buffer Revert Aid (Thermo Fisher Scientific). Transcriptional expression was quantified by RT-qPCR. Primers were designed using Primer3 software (v0.4.0 (24), Table S2). One PCR reaction contained 2 µl diluted cDNA (10 ng/µl), 3 µl H_2O PCR grade, 0.5 µl of each primer (4 µM), and 6 µl 2x buffer SensiFAST SYBR No-ROX mix (Bioline, Luckenwalde, Germany) and was carried out in triplicates using the LightCycler 96 (Roche, Basel, Switzerland). Amplicons were sequenced on an ABI 3130 Genetic Analyzer (Life Technologies GmbH, Darmstadt, Germany). The obtained sequences were blasted using NCBI BLAST tool to confirm sequence identity. The efficiency of amplification was calculated using LinRegPCR software (v2014.4; Academic Medical Centre, Amsterdam, Netherlands; (25)), yielding efficiency values between 1.81 and 1.87 (Table S2). Data were quantified by qbase software (Biogazelle, Gent, Belgium) using *hypoxanthine phosphoribosyltransferase 1* (HPRT1) and *ribosomal protein L0* (RPL0) as reference genes (M-value 0.297; V-value 0.118).

Western blot
Western blot analysis was performed to investigate the effects of heat stress on the expression of the TJP zonula occludens 1 (ZO-1) and claudin-1 in the jejunum. Mucosa tissue (30 mg) was homogenized in lysis buffer (pH 7.8) containing 50 mM Tris-HCl, 1 mM EDTA, 10 mM NaF, 1 % IGPEAL CA-630, 0.1 % Triton X100, 0.5 % deoxycholic acid (DCA) and 0.1 % SDS. Protein concentrations were measured using the Bradford kit (Thermo Fisher Scientific). Equal amounts of protein (25 µg) were separated by SDS-PAGE. Separated proteins were transferred to either a nitrocellulose (claudin-1: Whatman Protran BA 83, Dassel, Germany) or PVDF membrane (ZO-1: Carl Roth, Karlsruhe, Germany). Membranes were stained with Ponceau S to confirm equal loading, scanned and saved as image file. Membranes were blocked with 3 % bovine serum albumin (BSA) in Tris-buffered saline (TBS, pH 7.8) containing 0.1 % Tween 20 (TBST)

for 1 h and then incubated overnight with the following primary antibodies: ZO-1 (dilution 1:1,000; H-300 Sc-10804, Santa Cruz Biotechnology, Dallas, TX, USA) and claudin-1 (dilution 1:250; sc-166338, Santa Cruz Biotechnology). After incubation, membranes were washed 3x in TBST, incubated for 1 h at room temperature with the corresponding secondary antibody (goat anti rabbit IgG HRP, 1:10,000 dilution; sc-2004, Santa Cruz Biotechnology, Dallas, TX, USA or rabbit anti mouse IgG HRP, 1:10,000 dilution; AS09 627, Agrisera AB, Vännäs, Sweden) and washed again 3x in TBST. Chemiluminescent reagents (Pierce ECL Western Blotting Substrate, Thermo Fisher Scientific) were applied, and blots were exposed to hyperfilms (GE Healthcare, Chalfont St Giles, Buckinghamshire, UK). Hyperfilms and Ponceau S stained membranes were scanned and individual bands or lanes, respectively, were quantified using ImageJ (v1.49). Optical densities of immunostained bands were normalized to the corresponding Ponceau S stained lanes.

Cytokine ELISA

Potential effects of heat stress on the local immune status were investigated by quantifying interleukin-1β (IL-1β) and interleukin-4 (IL-4) in the jejunal mucosa. Sixty mg mucosa was homogenized in ice-cold phosphate buffered saline (PBS) containing: 0.4 M NaCl, 0.05 % Tween 20, 0.5 % BSA, 0.1 mM phenylmethylsulphonyl fluoride, 0.1 mM benzethonium chloride, 10 mM EDTA and 20 KI/ml aprotinin (Sigma-Aldrich). The homogenates were centrifuged at 10,000 rpm for 20 min at 4 °C and protein concentrations of the supernatant were determined using the Bradford method. The concentration of IL-1β and IL-4 in jejunum mucosa extracts was determined by capture ELISA performed on 96-well microtiter plates according to manufacturer's instructions (Bovine IL-1β ELISA, ESS0027, Bovine IL-4 ELISA, ESS0031; Thermo Fisher Scientific). Cytokine concentrations were calculated from a standard curve and expressed as pg/ml (Tecan SUNRISE 96-well microplate reader, Tecan, Männedorf, Switzerland). Data were normalized to total protein concentration (pg/mg) of the homogenate.

Jejunal inflammation and stress markers

Mucosa samples were analyzed for lysozyme activity using EnzChek Lysozyme Assay kit (Thermo Fisher Scientific) as previously described (7). The assay detection range was 0 to 500 U/ml and measurements were taken after 60 min of incubation. Tissue alkaline phosphatase was measured with a commercial kit (QuantiChrom Alkaline Phosphatase, BioAssay Systems, Hayward, CA, USA) according to the manufacturer´s instructions. Catalase activity measurement was performed as previously described (26) using a Catalase Assay kit (Cayman Chemicals, Ann Arbor, MI, USA). All concentrations determined were normalized to the total protein concentration (Bradford, Thermo Fisher Scientific) of the tissue homogenates.

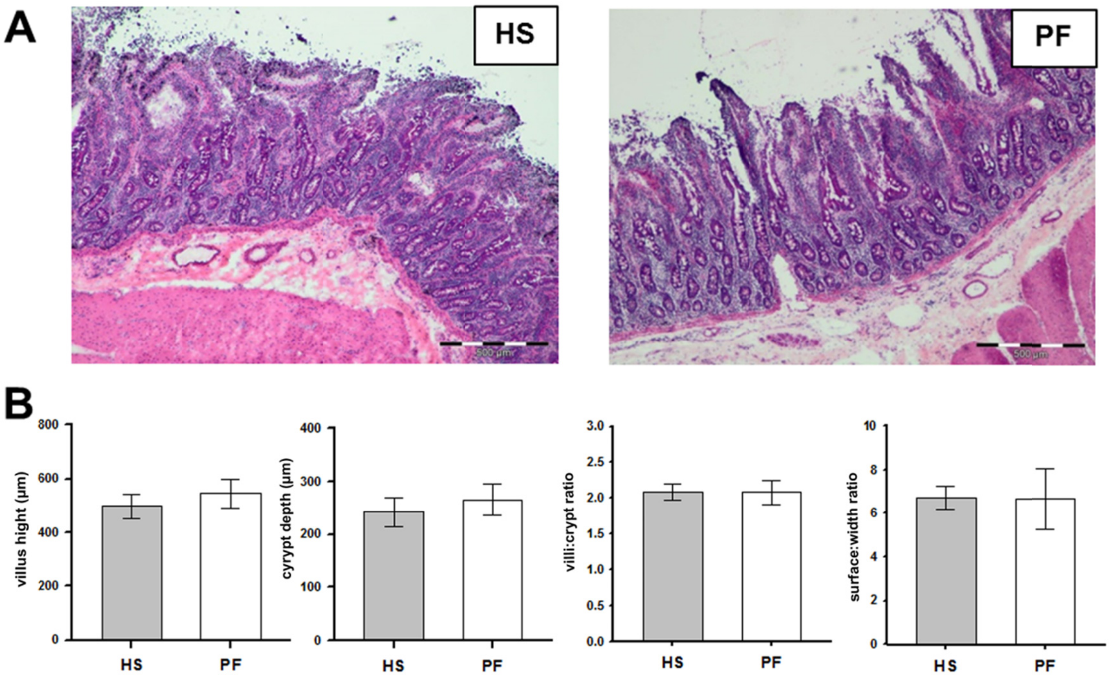

Fig. S1. Histological analysis of the jejunum from heat-stressed (HS) and pair-fed (PF) cows. (A) Representative image of the jejunum of a HS and a PF dairy cow, stained with hematoxylin/eosin (scale bar, 500 µm). (B) Morphometric analysis of villi hight, crypt depth, villi:crypt ratio and surface:width ratio. n = 5 (means ± SEM).

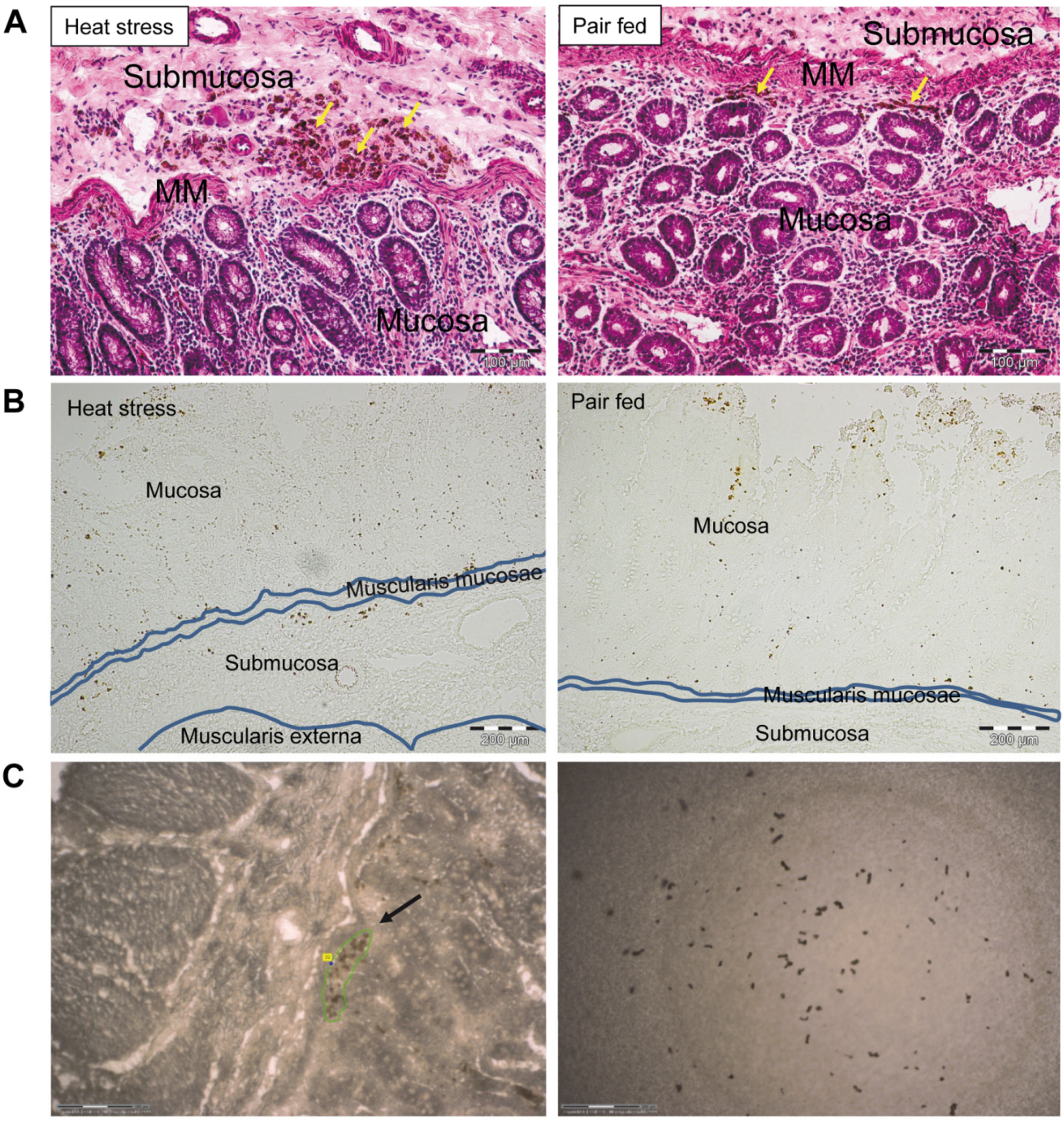

Fig. S2. Morphology of bovine jejunum. (A) Representative image with infiltrating cells (yellow arrows) in the submucosa or mucosa after four days of heat stress or pair feeding (hematoxylin/eosin, scale bar 100 µm). MM – Muscularis mucosae. (B) Representative bright field image of unstained jejunum of heat-stressed and pair-fed dairy cows (scale bar, 200 µm). (C) Visualization of infiltrating cells (arrow) in the jejunum of HS cows and collection with laser capture microdissection in an adhesive cap.

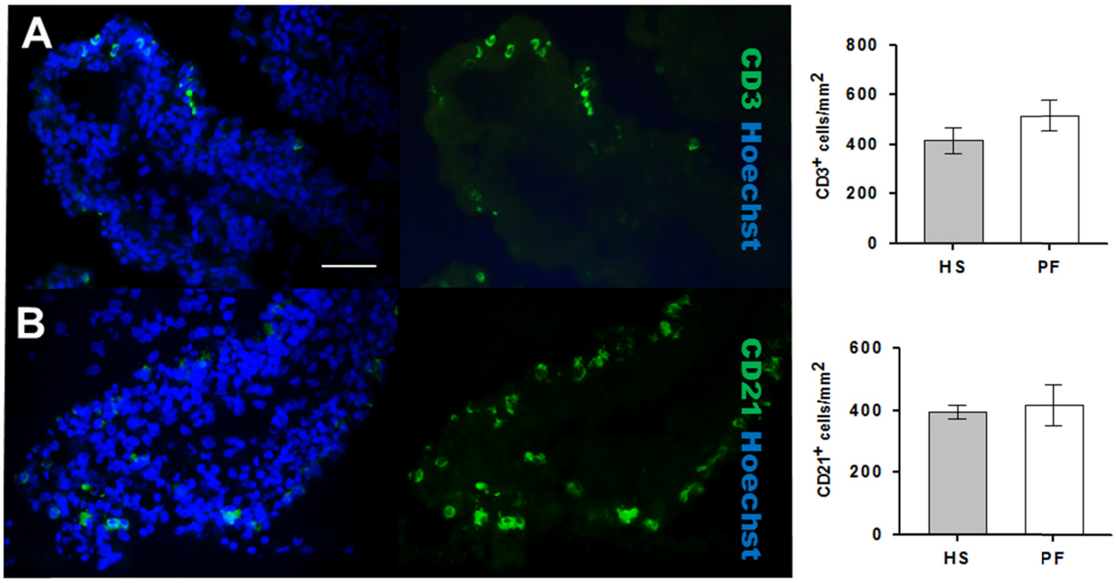

Fig. S3. Presence of CD3 positive B-cells and CD21 positive T-cells in the villi of the jejunum of heat-stressed (HS) and pair-fed (PF) cows. (A) CD3 fluorescence (green) in a representative jejunum section and number of CD3 positive cells per mm^2 of HS and PF dairy cows. (B) CD21 fluorescence (green) of a representative jejunum section and number of CD21 positive cells per mm^2 of HS and PF dairy cows. Scale bar 200 µm, n = 5 (means ± SEM).

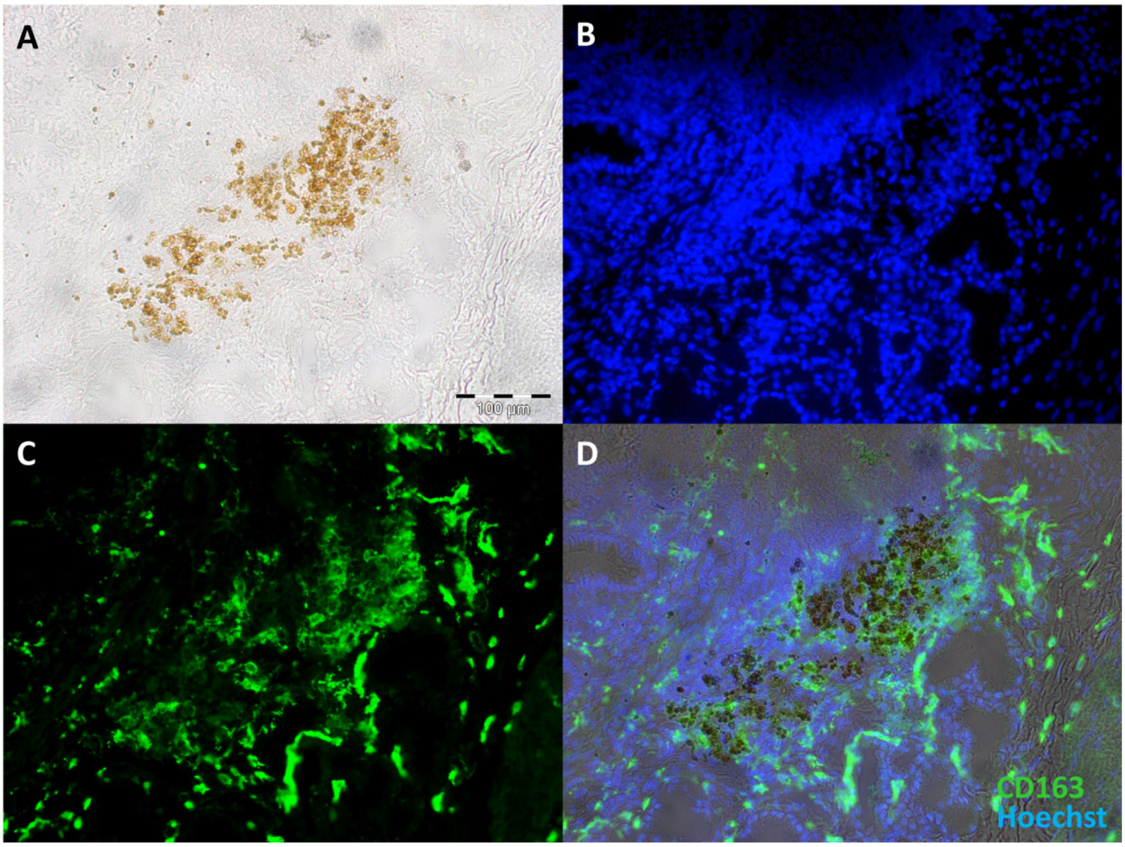

Fig. S4. Detection of CD163 positive macrophages in infiltrating cells of the jejunum of heat-stressed cows. (A) Brightfield image (scale bar, 100 µm), (B) nuclei stained with Hoechst 33258, (C) CD163 positive cells, (D) merged image (green – CD163, blue – Hoechst 33258).

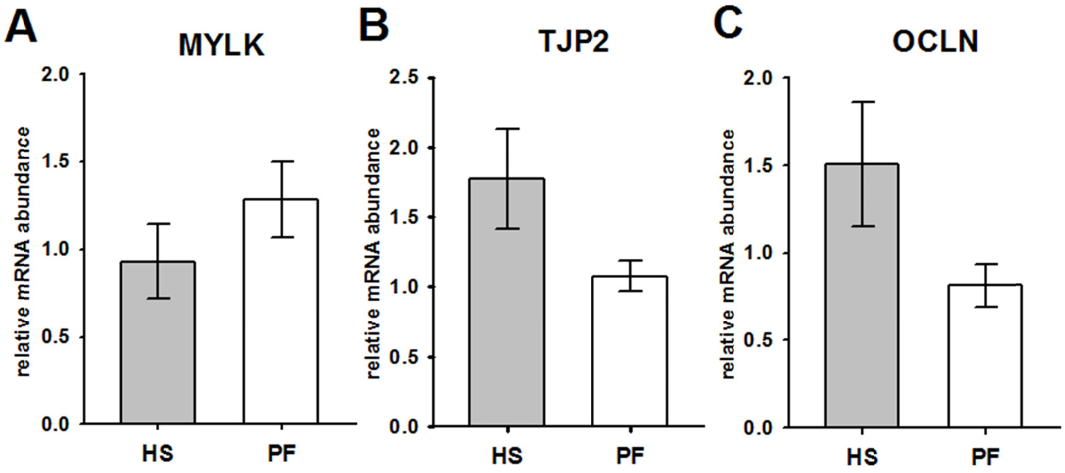

Fig. S5. Gene expression of tight junction proteins in the jejunal mucosa of heat-stressed (HS) and pair-fed (PF) cows. (A) *MYLK*, (B) *TJP2* and (C) *OCLN*. n = 5 (means ± SEM).

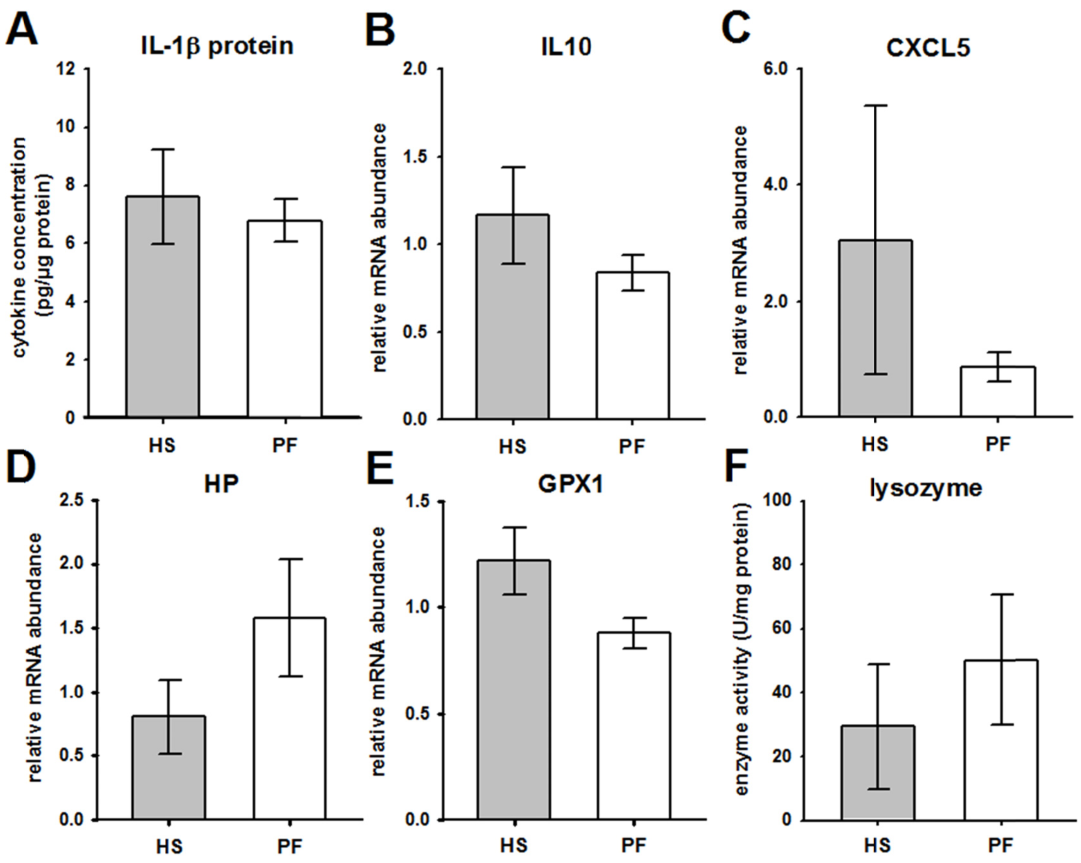

Fig. S6. Inflammation and oxidative stress markers in the jejunal mucosa of heat-stressed (HS) and pair-fed (PF) cows. (A) IL-1β protein abundance. mRNA abundance of (B) *IL10*, (C) *CXCL5,* (D) *HP*, (E) *GPX1* and (F) lysozyme activity in the jenunal mucosa of PF and HS cows. n = 5 (means ± SEM).

Table S1. Differential expression of marker genes specific for particular cell types or immune functions in transcriptomes of infiltrating cells in heat-stressed cows relative to whole jejunal mucosa of age-matched cows.

Gene name/ Ensembl ID	Surface marker/ Gene symbol	logFC	log CPM	q-value	Functions
**Macrophages**					
CD68 molecule, ENSBTAG00000000133	CD68	5.66	9.07	1.81E-38	expressed by human monocytes and tissue macrophages, clear cellular debris, promote phagocytosis, mediate recruitment and activation of macrophages
CD163 molecule, ENSBTAG00000019669	CD163	3.14	5.51	1.67E-12	expressed in monocytes and macrophages, innate immune sensor for bacteria and inducer of local inflammation
CD14 molecule, ENSBTAG00000015032	CD14	2.74	5.99	7.38E-14	expressed on monocytes/macrophages, coreceptor for bacterial LPS, cooperates with other proteins to mediate innate immune response to bacterial LPS
Mannose Receptor C-Type 1, ENSBTAG00000038048	CD206, MRC1	2.18	8.03	4.44E-10	mediates endocytosis of glycoproteins of pathogenes by macrophages
ATP-binding cassette transporter 9, ENSBTAG00000006896	ABCA9	4.56	5.32	1.43E-29	transcriptional expression is induced during monocyte differentiation into macrophages
Glycoprotein nonmetastatic melanoma B, ENSBTAG00000000604	GPNMB	8.29	11.48	4.35E-27	expressed in macrophages, negative feedback regulator of pro-inflammator response
Carboxypeptidase M, ENSBTAG00000000604	CPM	4.04	7.79	4.26E-07	expression is associated with monocyte to macrophage differentiation
Fc Fragment Of IgG Receptor IIa , ENSBTAG00000038124	CD32, FCGR2A	2.49	3.18	0.046	expressed by macrophages and neutrophils, phagocytosis and clearing of immune complexes
Fc Fragment of IgG Receptor IIa, ENSBTAG00000021842	CD32, FCGR2B	2.88	3.91	6.22E-23	involved in a variety of effector and regulatory functions such as phagocytosis of immune complexes and modulation of antibody production by B cells
**Myeloid cells (Macrophages and dendritic cells)**					

Integrin Subunit Alpha M, ENSBTAG00000047238	CD11b, ITGAM	1.17	7.53	n.s.	implicated in various adhesive interactions of monocytes, macrophages and granulocytes and in mediating uptake of complement-coated particles
Signal Regulatory Protein Alpha, ENSBTAG00000007213	CD172a, SIRPA	1.60	7.52	1.17E-03	negative regulation of receptor tyrosine kinase-coupled cellular responses induced by cell adhesion, growth factors or insulin, mediates negative regulation of phagocytosis, mast cell activation and dendritic cell activation
CD200 Receptor 1, ENSBTAG00000001235	CD200R1	1.64	4.91	0.042	limits inflammation by inhibiting the expression of proinflammatory molecules e.g. TNF-alpha, interferons, inducible NO synthase in response to selected stimuli
**Dendritic cells**					
Lymphocyte Antigen 75, ENSBTAG00000017797	CD205, LY75	-1.19	7.89	n.s.	endocytic receptor, direct captured antigens from extracellular space to specialized antigen-processing compartment, causes reduced B lymphocyte proliferation
**Monocytes**					
CD9 molecule, ENSBTAG00000014764	CD9	1.0	6.82	n.s.	involved in differentiation, adhesion and signal transduction
Fc fragment of IgA receptor, ENSBTAG00000021647	CD89, FCAR	-10.84	3.28	3.13E-09	on the surface of neutrophils, monocytes, macrophages and eosinophils, mediates immunologic responses to pathogens.
**B cells**					
CD40 ligand, ENSBTAG00000017843	IGM, CD40LG	0.54	3.81	n.s.	costimulates T cell proliferation and cytokine production, cross-linking on T cells; generates a costimulatory signal which enhances the production of IL4 and IL10
Fc Fragment of IgE Receptor II, ENSBTAG00000004270	CD23, FCER2	2.55	3.35	n.s.	essential roles in B cell growth and differentiation and regulation of IgE production
Complement C3d Receptor 2, ENSBTAG00000038496	CD21, CR2	3.98	5.48	4.98E-21	functions as a receptor for Epstein-Barr virus (EBV) binding on B and T lymphocytes
CD19 molecule, ENSBTAG00000032122	CD19	0.21	2.88	n.s.	assembles with the antigen receptor of B lymphocytes
**T cells**					
CD4 molecule, ENSBTAG00000003255	CD4	1.54	5.12	n.s.	in T lymphocytes, B cells, macrophages and granulocytes; triggers differentiation of monocytes into mature functional macrophages
CD8A molecule, ENSBTAG00000021141	CD8A	-1.27	4.37	n.s.	mediates efficient cell-cell interactions within the immune system

CD3E molecule, ENSBTAG00000015710	CD3E	-0.89	6.67	n.s.	essential role in adaptive immune response
Dipeptidyl Peptidase 4, ENSBTAG00000015330	CD26, DPP4	-2.11	5.80	n.s.	involved in costimulatory signal essential for T-cell receptor (TCR)-mediated T-cell activation
Cytotoxic T-Lymphocyte Associated Protein 4, ENSBTAG00000013170	CD152, CTLA4	0.81	2.01	n.s.	transmits an inhibitory signal to T cells
Interleukin 2 Receptor Subunit Alpha, ENSBTAG00000020892	CD25, IL2RA	-0.38	4.16	n.s.	involved in the regulation of immune tolerance by controlling regulatory T cells (TREGs) activity
Forkhead Box P3, ENSBTAG00000013279	FOXP3	n.s	n.s	n.s.	development and inhibitory function of TREGs
**Natural killer cells**					
Fc Fragment of IgG Receptor IIIa, ENSBTAG00000002096	CD16, FCGR3A	1.48	7.68	n.s.	mediates antibody-dependent cellular cytotoxicity and other antibody-dependent responses, such as phagocytosis
Natural Cytotoxicity Triggering Receptor 1, ENSBTAG00000045529	CD335, NCR1	-7.07	0.057	n.s.	may contribute to the increased efficiency of activated natural killer cells to mediate tumor cell lysis
**Epithelial cells**					
Aminopeptidse N, ENSBTAG00000016881	CD13, ANPEP	-11.9	9.36	9.61E-82	involved in metabolism of regulatory peptides in intestinal, renal tubular epithelial cells, macrophages, granulocytes, and synaptic membranes, mediates phagocytosis

#Function according to Gene Cards, http://www.genecards.org/

Table S2. Primer sequences and RT-qPCR conditions.

Gene		Primer sequence (5' to 3')	GenBank accession no.	Product Size (base pairs)	Annealing (°C / s)	Efficiency
CAT	forward	TCACTCAGGTGCGGACTTTC	NM_001035386	162	60	1.823
	reverse	GGATGCGGGAGCCATATTCA			60	
CLDN1	forward	TTCGACTCCTTGCTGAATCTG	NM_001001854.2	84	60	1.821
	reverse	GGCTATTAGTCCCAGCAGGATG			60	
CXCL5	forward	GCAACCTGCCCTATAACGGT	AC_000163.1	106	60	1.851
	reverse	GGATGAATTCCCGGTGTGGT			60	
GPX1	forward	CTTCCCCTGCAACCAGTTTG	X13684.1	62	62	1.865
	reverse	GGCAATTCAGGATCTCCTCGTT			62	
HP	forward	GGCCCCCGAGATTGCTAATA	NM_001040470.2	172	60	1.86
	reverse	CTCTGGGCAGCTGTCATCTT			60	
HPRT1	forward	TACTGCTACTGTGTGCTTAGG	NM_001034035.2	111	60	1.841
	reverse	CTACTGAAACACTGGCGGGAC			60	
IL10	forward	CTTTAAGGGTTACCTGGGTTGC	NM_174088	262	60	1.809
	reverse	CTCACTCATGGCTTTGTAGACAC			60	
IL4	forward	TCAAAACGCTGAACATCCTCAC	NM_173921.2	70	60	1.844
	reverse	GCAAAGACGTCTGCTACAGG			60	
IL6	forward	ACCCCAGGCAGACTACTTCT	NM_173923	213	60	1.878
	reverse	GCAAATCGCCTGATTGAACCC			60	
MYCK	forward	AGTACATCCACAAGCAGGGC	NM_176636.2	240	60	1.845
	reverse	GGATGTAGCAGATGACCCCG			60	
OCLN	forward	CCTTTTGAAAGTCCACCTCCTTAT	NM_001082433.2	70	60	1.845
	reverse	TGTCATTGCTTGGTGTGTAGT			60	
RPL0	forward	CAACCCTGAAGTGCTTGACAT	NM_001012682	227	60	1.818
	reverse	AGGCAGATGGATCAGCCA			60	
SOD1	forward	AAGGCCGTGTGCGTGCTGAA	NM_174615.2	246	62	1.841
	reverse	CAGGTCTCCAACATGCCTCT			62	
TNFA	forward	AGAGGGAAGAGCAGTCCCCAG	NM_173966.3	181	60	1.813
	reverse	TTCACACCGTTGGCCATGAG			60	
TJP1	forward	AGAAAGATGTTTATCGTCGCATCGT	AC_000178.1	84	60	1.811
	reverse	ATTCCTTCTCATATTCAAAATGGGTTCTGA			60	
TJP2	forward	TGCTCCATTCATTTGCGGTTC	NM_001102482.1	70	58	1.811
	reverse	GGCCTCTTGACCACAATGG			58	
TNFA	forward	AGAGGGAAGAGCAGTCCCCAG	NM_173966.3	181	60	1.813
	reverse	TTCACACCGTTGGCCATGAG			60	

Additional data tables (separate files)

Dataset S1. Analysis of FPKM values for all genes listed in the Ensembl annotation v87 in infiltrating cells of heat-stressed cows.

Dataset S2. Comparison between the 215 differently expressed genes within NuGen Ovation SoLo libraries (infiltrating cells of heat-stressed cows vs. whole jejunum control mucosa) and the edgeR-based calculation of differential expression between infiltrating cells in HS cows and the whole jejunum mucosa of age-matched cows.

Dataset S3. Differential transcriptome analysis by edgeR between infiltrating cells in jejunum in response to heat stress and whole jejunal mucosa from age-matched control cows.

Dataset S4. Full list of canonical pathways significantly enriched with genes significantly higher expressed in infiltrating cells in heat-stressed cows than in the whole jejunum mucosa of age-matched control cows.

Dataset S5. Full list of upstream regulators with significant overlap with genes significantly higher expressed in infiltrating cells in heat-stressed cows than in the whole jejunum mucosa of age-matched control cows.

References

1. Basirico L*, et al.* (2011) Cellular thermotolerance is associated with heat shock protein 70.1 genetic polymorphisms in Holstein lactating cows. *Cell stress Chaperones* 16(4):441-448.
2. Baumgard LH & Rhoads RP (2012) Ruminant Nutrition Symposium: ruminant production and metabolic responses to heat stress. *J Anim Sci* 90(6):1855-1865.
3. Lamp O*, et al.* (2015) Metabolic Heat Stress Adaption in Transition Cows: Differences in Macronutrient Oxidation between Late-Gestating and Early-Lactating German Holstein Dairy Cows. *PloS One* 10(5):e0125264.
4. Vanselow J, Vernunft A, Koczan D, Spitschak M, & Kuhla B (2016) Exposure of Lactating Dairy Cows to Acute Pre-Ovulatory Heat Stress Affects Granulosa Cell-Specific Gene Expression Profiles in Dominant Follicles. *PloS One* 11(8):e0160600.
5. Hammon HM*, et al.* (2018) Different milk diets have substantial effects on the jejunal mucosal immune system of pre-weaning calves, as demonstrated by whole transcriptome sequencing. *Sci Rep* 8(1):1693.
6. Weikard R, Goldammer T, Brunner RM, & Kuehn C (2012) Tissue-specific mRNA expression patterns reveal a coordinated metabolic response associated with genetic selection for milk production in cows. *Physiol Genomics* 44(14):728-739.
7. Pearce SC*, et al.* (2013) Heat stress and reduced plane of nutrition decreases intestinal integrity and function in pigs. *J Anim Sci* 91(11):5183-5193.
8. Albrecht E, Gotoh T, Ebara F, Wegner J, & Maak S (2011) Technical note: Determination of cell-specific gene expression in bovine skeletal muscle tissue using laser microdissection and reverse-transcription quantitative polymerase chain reaction. *J Anim Sci* 89(12):4339-4343.

9. Weikard R, Goldammer T, Eberlein A, & Kuehn C (2009) Novel transcripts discovered by mining genomic DNA from defined regions of bovine chromosome 6. *BMC Genomics* 10:186.
10. Andrews S (2010) FastQC: a quality control tool for high throughput sequence data. ed http://www.bioinformatics.babraham.ac.uk/projects/fastqc/.
11. Martin M (2011) Cutadapt removes adapter sequences from high-throughput sequencing reads. *EMBnet.journal* 17(1):10-12.
12. Kim D, Langmead B, & Salzberg SL (2015) HISAT: a fast spliced aligner with low memory requirements. *Nat Methods* 12(4):357-360.
13. Robinson JT*, et al.* (2011) Integrative genomics viewer. *Nat Biotechnology* 29(1):24-26.
14. Thorvaldsdottir H, Robinson JT, & Mesirov JP (2013) Integrative Genomics Viewer (IGV): high-performance genomics data visualization and exploration. *Briefings Bioinformatics* 14(2):178-192.
15. Liao Y, Smyth GK, & Shi W (2014) featureCounts: an efficient general purpose program for assigning sequence reads to genomic features. *Bioinformatics* 30(7):923-930.
16. McCarthy DJ, Chen Y, & Smyth GK (2012) Differential expression analysis of multifactor RNA-Seq experiments with respect to biological variation. *Nucleic Acids Res* 40(10):4288-4297.
17. Kramer A, Green J, Pollard J, Jr., & Tugendreich S (2014) Causal analysis approaches in Ingenuity Pathway Analysis. *Bioinformatics* 30(4):523-530.
18. Olsen J*, et al.* (1988) Complete amino acid sequence of human intestinal aminopeptidase N as deduced from cloned cDNA. *FEBS letters* 238(2):307-314.
19. Consortium GT (2013) The Genotype-Tissue Expression (GTEx) project. *Nat Genetics* 45(6):580-585.
20. Fries PN, Popowych YI, Guan LL, & Griebel PJ (2011) Age-related changes in the distribution and frequency of myeloid and T cell populations in the small intestine of calves. *Cell Immunol* 271(2):428-437.
21. Menge C*, et al.* (2015) Bovine macrophages sense Escherichia coli Shiga toxin 1. *Innate Immun* 21(6):655-664.
22. Fuertes M*, et al.* (2015) Immunohistochemical study and mRNA cytokine profile of the local immune response in cattle naturally infected with Calicophoron daubneyi. *Vet Parasitol* 214(1-2):178-183.
23. Corripio-Miyar Y*, et al.* (2015) Phenotypic and functional analysis of monocyte populations in cattle peripheral blood identifies a subset with high endocytic and allogeneic T-cell stimulatory capacity. *Vet Res* 46:112.
24. Ye J*, et al.* (2012) Primer-BLAST: a tool to design target-specific primers for polymerase chain reaction. *BMC Bioinformatics* 13:134.
25. Ruijter JM*, et al.* (2013) Evaluation of qPCR curve analysis methods for reliable biomarker discovery: bias, resolution, precision, and implications. *Methods* 59(1):32-46.
26. Liu F*, et al.* (2016) Selenium and Vitamin E together improve intestinal epithelial barrier function and alleviate oxidative stress in heat stressed pigs. *Exp Physiol* 101(7):801-810.
